# Development of a Triplex qPCR Assay Based on the TaqMan Probe for the Detection of *Haemophilus parasuis*, *Streptococcus suis* Serotype 2 and *Pasteurella multocida*

**DOI:** 10.3390/microorganisms12102017

**Published:** 2024-10-05

**Authors:** Kaili Li, Yu Zhang, Tingyu Luo, Changwen Li, Haibo Yu, Wei Wang, He Zhang, Hongyan Chen, Changyou Xia, Caixia Gao

**Affiliations:** State Key Laboratory for Animal Disease Control and Prevention, Heilongjiang Provincial Key Laboratory of Laboratory Animal and Comparative Medicine, National Poultry Laboratory Animal Resource Center, Harbin Veterinary Research Institute, Chinese Academy of Agricultural Sciences, Harbin 150069, China; likailiv@163.com (K.L.); zhangyu99116@163.com (Y.Z.); 18328023988@163.com (T.L.); lichangwen@caas.cn (C.L.); yuhaibo@caas.cn (H.Y.); wangwei02@caas.cn (W.W.); zhanghe01@caas.cn (H.Z.); chenhongyan@caas.cn (H.C.)

**Keywords:** porcine respiratory disease, *Haemophilus parasuis*, *Streptococcus suis* serotype 2, *Pasteurella* *multocida*, real-time quantitative polymerase chain reaction

## Abstract

Porcine respiratory disease is a significant economic problem for the global swine industry. *Haemophilus parasuis* (*H. parasuis*), *Streptococcus suis* (*S. suis*), and *Pasteurella multocida* (*P. multocida*) are three important pathogenic bacteria of the swine respiratory tract. Notably, the three pathogens not only frequently manifest as mixed infections, but their striking clinical similarities also present difficulties for pig populations in terms of disease prevention and treatment. Thus, we developed a triplex real-time quantitative polymerase chain reaction (qPCR) assay based on a TaqMan probe for the detection of *H. parasuis*, *S. suis* serotype 2, and *P. multocida*. Primers and probes were designed to target the conserved regions of the *H. parasuis OmpP2* gene, the *S. suis* serotype 2 *gdh* gene, and the *P. multocida Kmt1* gene. By optimizing the reaction system and conditions, a triplex qPCR method for simultaneous detection of *H. parasuis*, *S. suis* serotype 2, and *P. multocida* was successfully established. The amplification efficiencies of the standard curves for all three pathogens were found to be highly similar, with values of 102.105% for *H. parasuis*, 105.297% for *S. suis* serotype 2, and 104.829% for *P. multocida*, and all R^2^ values achieving 0.999. The specificity analysis results showed that the triplex qPCR method had a strong specificity. The sensitivity test results indicated that the limit of detection can reach 50 copies/μL for all three pathogens. Both intra- and inter-assay coefficients of variation for repeatability were below 1%. This triplex qPCR method was shown to have good specificity, sensitivity, and reproducibility. Finally, the triplex qPCR method established in this study was compared with the nested PCR as recommended by the Chinese national standard (GB/T34750-2017) for *H. parasuis*, the PCR as recommended by the Chinese national standard (GB/T 19915.9-2005) for *S. suis* serotype 2, and the PCR as recommended by the Chinese agricultural industry standard (NY/T 564-2016) for *P. multocida* by detecting the same clinical samples. Both methods are reasonably consistent, while the triplex qPCR assay was more sensitive. In summary, triplex qPCR serves not only as a rapid and accurate detection and early prevention method for these pathogens but also constitutes a robust tool for microbial quality control in specific pathogen-free pigs.

## 1. Introduction

Porcine respiratory disease seriously hinders the global development of the swine industry. *Haemophilus parasuis* (*H. parasuis*), *Streptococcus suis* (*S. suis*), and *Pasteurella multocida* (*P. multocida*) are three pathogens that are closely associated with porcine respiratory disease [1,2]. *H. parasuis* is a Gram-negative bacterium with a genome size of approximately 2.13–2.49 Mb, including the *outer membrane protein* (*omp*) *P2* gene, which has been shown to be highly homologous [3]. It serves as the etiological agent of Glässer’s disease, manifesting as polyserositis in piglets and pneumonia in adults. It was first described by Glässer in 1910 and isolated by Schermer and Ehrlich in 1922 [4,5]. *H. parasuis* is a prevalent bacterium of the upper respiratory tract of swine, encompassing strains with varying degrees of virulence [6]. To date, fifteen serotypes have been identified, with serotypes 4 and 5 being the most prevalent in China [6]. In immunocompromised animals under stressful conditions, *H. parasuis* transforms into a pathogen that elicits Glässer’s disease, which is typically characterized by meningitis, polyarthritis, fibrinous polyserositis, and sometimes acute pneumonia and septicemia [7]. *S. suis*, a facultative anaerobic Gram-positive bacterium, colonizes the respiratory tract of healthy pigs, specifically the tonsils and nasal cavity, with nearly 100% colonization rates in swine tonsils [8]. Under certain conditions, *S. suis* infections manifest clinically as arthritis, pneumonia, and endocarditis, with severe cases progressing rapidly to septic shock and potential fatality [9,10]. Currently, 29 recognized serotypes exist, with serotype 2 being the most virulent and frequently isolated, posing a significant public health threat [11,12]. *S. suis* serotype 2 not only possesses the ability to survive under adverse conditions but also enhances its survival strategy by forming biofilms [13]. Among its numerous virulence factors, glyceraldehyde-3-phosphate dehydrogenase (gdh), which is located on the cell wall, stands out particularly due to its high amino acid sequence homology, ranging from 99% to 100%, making it the most conserved gene [14]. *P. multocida*, a facultative anaerobic Gram-negative bacterium, resides in the oral, upper respiratory, and gastrointestinal microbiomes of animals [15,16]. *P. multocida* is classified into five serotypes (A, B, D, E, and F) based on capsular antigens. In China, serotypes A and D of *P. multocida* are the most prevalent serotypes in swine, which typically cause pneumonia and progressive atrophic rhinitis in grower-finisher pigs, resulting in growth retardation and diminished efficiency of feed utilization, causing significant economic losses in the pig industry [17,18]. Notably, despite the extensive nature and complex variability of the *P. multocida* genome, the *Kmt1* gene exhibits a high degree of conservation between serotypes A and D [19].

*H. parasuis*, *S. suis* serotype 2, and *P. multocida* commonly colonize the swine upper respiratory tract, but disease outbreaks are triggered by specific conditions such as co-infections, environmental alterations, and pharmacological treatments [20,21]. Due to the similarity of clinical symptoms caused by the three pathogens, co-infections aggravate the difficulties in differential diagnosis and disease prevention. Despite the formulation of national and industrial standards in China for detecting these pathogens, including polymerase chain reaction (PCR), nested PCR, and real-time quantitative PCR (qPCR), most current methods are limited to single pathogen detection. Consequently, the development of more efficient, multi-pathogen detection methods is imperative.

Real-time quantitative PCR (qPCR) is a potent and user-friendly technology that offers a heightened level of specificity and sensitivity compared to conventional bacterial culture and conventional PCR methods. It circumvents the limitations of slow turnaround times and complex post-amplification manipulations, eliminating the need for such procedures [22,23]. The TaqMan qPCR assay allows for the simultaneous detection of multiple distinct pathogens within a single reaction vessel. This is achieved through the utilization of specifically designed probes for each target DNA, with each probe being labeled with a distinct fluorescent signal, thereby enabling the differentiation of various pathogens. Additionally, the capability for high-throughput detection significantly enhances detection efficiency, making it an ideal methodology for multi-pathogen screening in contemporary laboratory settings [23,24]. The majority of existing fluorescent detection techniques for *H. parasuis*, *S. suis* serotype 2, and *P. multocida* are predominantly focused on single or dual fluorescent probe assays. Consequently, it is essential to develop a triplex TaqMan qPCR method that can simultaneously target the three pathogens.

In this study, we designed primers and probes targeting conserved regions of the *OmpP2* gene of *H. parasuis*, the *gdh* gene of *S. suis* serotype 2, and the *Kmt1* gene of *P. multocida*. A triplex qPCR assay was developed that can simultaneously detect *H. parasuis*, *S. suis* serotype 2, and *P. multocida*. The method not only provides an efficient and sensitive method for disease diagnosis and epidemiological studies but also serves as a tool for microbiological quality control in Specific Pathogen Free (SPF) pigs.

## 2. Materials and Methods

### 2.1. Pathogenic Nucleic Acids and Clinical Samples

Genomic DNA of *H. parasuis*, *S. suis* serotype 2, *P. multocida*, *Mycoplasma hyopneumoniae* (*M. hyopneumoniae*), *Mycoplasma hyorhinis* (*M. hyorhinis*), *Actinobacillus pleuropneumoniae* (*A. pleuropneumoniae*), porcine circovirus type 2 (PCV 2), pseudorabies virus (PRV), genomic cDNA of porcine reproductive and respiratory syndrome virus (PRRSV), and swine influenza virus (SIV) were stored in the State Key Laboratory for Animal Disease Control and Prevention, Harbin Veterinary Research Institute, Chinese Academy of Agricultural Sciences. In addition, 54 throat swab samples and 54 nasal swab samples from pigs, collected from farms in Heilongjiang Province, were kindly provided by another laboratory of the Harbin Veterinary Research Institute, Chinese Academy of Agricultural Sciences. It is imperative to underscore that no additional harm or intervention was imposed on the animals involved in this study. Given the nature of our research, the Institutional Review Board of the Harbin Veterinary Research Institute has determined that this study is exempt from the requirement for ethical review or approval.

### 2.2. Primers and TaqMan Probes

For primers and TaqMan probes, a varying number of representative sequences (17 for the *H. parasuis OmpP2* gene, 19 for the *S. suis* serotype 2 *gdh* gene, and 10 for the *P. multocida Kmt1* gene) were obtained from the GenBank database. These target gene sequences were aligned using MegAlign of Lasergene software (version 7.1.0.44) [25] to identify highly conserved regions. Three primer pairs and corresponding TaqMan probes were designed to target the conserved regions of the *H. parasuis OmpP2* (GenBank accession no. GU323691), *S. suis* serotype 2 *gdh* (GenBank accession no. CP018908), and *P. multocida Kmt1* (GenBank accession no. AY225342) using Primer Express software (version 3.0.1) [26]. The specificity of the primer pairs and probes was further confirmed through the utilization of the Blast tool provided by the National Center for Biotechnology Information (NCBI). For triplex detection, the probes were labeled with the different 5′-reporting dyes (FAM, VIC, and Cy5) and the corresponding 3′-quenchers (MGB, BHQ1, and BHQ2). The nested PCR recommended by the Chinese national standard (GB/T34750-2017) [27] for *H. parasuis*, the PCR recommended by the Chinese national standard (GB/T 19915.9-2005) [28] for *S. suis* serotype 2, and the PCR recommended by the Chinese agricultural industry standard (NY/T 564-2016) [29] for *P. multocida* were used to verify the accuracy of the triplex qPCR. The details of the primer pairs and probes are listed in Table 1.

### 2.3. Preparation of Standard Plasmid

**Figure 1:**
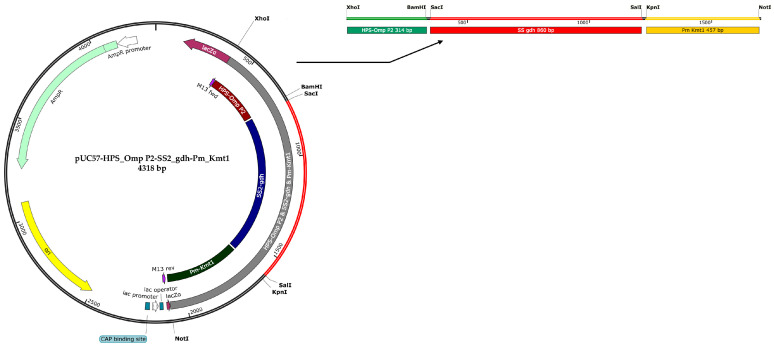
A recombinant plasmid was constructed, containing three conserved gene fragments: *H. parasuis OmpP2* (314 bp), *S. suis* serotype 2 *gdh* (860 bp), and *P. multocida Kmt1* (457 bp). Specific restriction enzyme cutting sites (XhoI and BamHI for *H. parasuis OmpP2*, SacI and SalI for *S. suis* serotype 2 *gdh*, KpnI and NotI for *P. multocida Kmt1*) were placed on both ends of each fragment.

A synthetic gene fragment, encompassing partial sequences of the *H. parasuis OmpP2* gene, the *S. suis* serotype 2 *gdh* gene, and the *P. multocida Kmt1* gene, was constructed by Sangon Biotech Co., Ltd. (Shanghai, China). This fragment was subsequently inserted into the pUC57 cloning vector, resulting in a standard plasmid named pUC57-HPS_OmpP2-SS2_gdh-Pm_Kmt1 (Figure 1), which will be utilized for further analysis. The sequence of the synthetic gene fragment is provided in Table S1. The concentration of the standard plasmid was measured using a Nanodrop spectrophotometer (Thermo Fisher Scientific, Waltham, MA, USA), and the formula for calculating the copy number of the standard plasmid is: Copies/μL = (A260 (ng/μL) × 10^−9^ × 6.02 × 10^23^)/(DNA length × 650). The standard plasmid was serially diluted 10-fold using EASY Dilution (Takara, Dalian, China) to establish a concentration gradient ranging from 1 × 10^8^ to 1 × 10^1^ copies/μL, which was then stored at −20 °C for subsequent experiments.

### 2.4. Establishment and Optimization of the Triplex qPCR System

The triplex qPCR reaction system (20 μL) was established according to the manufacturer’s instructions, comprising 10 μL of 2 × Premix Ex Taq (TaKaRa, Dalian, China), 0.4 μL of each primer (10 μM), 0.8 μL of each probe (10 μM), 0.4 μL of ROX reference dye, and 3 μL of template. The remaining volume was supplemented with nuclease-free H_2_O. The amplification parameters for the triplex qPCR system were established as follows: an initial denaturation step at 95 °C for 30 s, followed by 40 cycles consisting of denaturation at 95 °C for 5 s and annealing/extension at 60 °C for 31 s. Upon the completion of the reaction, the Ct value was automatically calculated by the system.

To optimize the reaction system, we explored various volumes of primers (applied concentration of 10 µM) and probes (applied concentration of 10 µM). The primer volume was optimized between 0.3 μL and 0.7 μL, resulting in a final concentration range of 150–350 nM, while the probe volume was optimized between 0.5 μL and 0.8 μL, yielding a final concentration range of 250–400 nM. 1 × 10^7^ copies/μL of the standard plasmid was used as a template for optimization. Ultimately, the fluorescence intensity and Ct value for each primer and probe concentration were compared to determine the optimal quantities. To further optimize the reaction efficiency, the annealing temperature was optimized by testing three temperatures (56 °C, 58 °C, and 60 °C), and the fluorescence intensity and Ct value were compared at each gradient.

### 2.5. Establishment of a Standard Curve

Using the final reaction conditions and protocol, a triplex qPCR standard curve was established using a 10-fold serial dilution of a standard plasmid with copy numbers ranging from 1 × 10^8^ to 1 × 10^2^ copies/μL as templates. A linear regression analysis was performed on the Ct values and the logarithm of the plasmid copy numbers. Seven-point standard curves were generated for *H. parasuis*, *S. suis* serotype 2, and *P. multocida*.

### 2.6. Specificity of the Triplex qPCR

In order to investigate the specificity of the triplex qPCR detection method, the genome DNA of *H. parasuis*, *S. suis* serotype 2, *P. multocida*, *M. hyopneumoniae*, *M. hyorhinis*, *A. pleuropneumoniae*, PCV2, PRV, the genome cDNA of PRRSV, and SIV were detected by the final reaction system. Additionally, 1 × 10^7^ copies/µL of the standard plasmid and nuclease-free H_2_O were used as positive and negative controls, respectively.

### 2.7. Sensitivity of the Triplex qPCR

To assess the sensitivity of the triplex qPCR, standard plasmids were serially diluted 10-fold to final concentrations ranging from 1 × 10^8^ to 1 × 10^1^ copies/µL, serving as templates to determine the limit of detection (LoD). Each reaction was replicated three times within a single test. The lowest copy number of the standard plasmid that could be consistently detected, based on the Ct values, was designated as the presumptive LoD. This presumptive LoD was refined through fine-tuning. Using the fine-tuned plasmids as templates, the tests were repeated 24 times to establish the definitive LoD. Although the MIEQ guidelines state that at least 95% probability of the repetitions is commonly used to determine LoD, they also indicate that in practice, this ‘standard’ is variable [30]. In order to ensure true and stable sensitivity in this study, the LoD was set as the minimum concentration of the standard plasmid that yielded positive results in at least 80% of the repetitions.

### 2.8. Repeatability of the Triplex qPCR

To assess the repeatability of the triplex qPCR, serial 10-fold dilutions of standard plasmids ranging from 1 × 10^6^ to 1 × 10^3^ copies/μL were utilized. Triplicate analyses of each dilution were performed on the same day to determine intra-assay variability. Additionally, three independent experiments were conducted by two distinct operators on separate days to assess inter-assay variability. In both intra- and inter-assay experiments, the coefficient of variation (CV) [30] of the Ct values across different concentration samples was calculated to estimate repeatability according to the following formula:

$$CV=\left(\frac{Standard\;Deviation}{Mean}\right)\times 100\%$$

### 2.9. Detection of Clinical Samples by Triplex qPCR

Genomic DNA was extracted from 54 throat swab samples and 54 nasal swab samples using a bacterial genomic DNA extraction kit (Tiangen Biotech Co., Ltd., Beijing, China). The DNA samples were tested using the triplex qPCR method. To validate the clinical performance, the same samples were analyzed by nested PCR as recommended by the Chinese national standard (GB/T34750-2017) [27] for *H. parasuis*, PCR as recommended by the Chinese national standard (GB/T 19915.9-2005) [28] for *S. suis* serotype 2, and PCR as recommended by the Chinese agricultural industry standard (NY/T 564-2016) [29] for *P. multocida*. The genomic DNA of *H. parasuis*, *S. suis* serotype 2, and *P. multocida* were used as positive controls, while nuclease-free H_2_O was utilized as a negative control. The reaction system and protocol for nested PCR of *H. parasuis* (GB/T34750-2017) [27], PCR of *S. suis* serotype 2 (GB/T 19915.9-2005) [28], and PCR of *P. multocida* (NY/T 564-2016) [29] are detailed in Table S2, respectively.

### 2.10. Statistical Analysis

The sequence alignments of *H. parasuis*, *S. suis* serotype 2, and *P. multocida* were performed using MegAlign of Lasergene software (version 7.1.0.44). The specific primers and probes were designed using Primer Express software (version 3.0.1). We used the Blast tool provided by the NCBI to verify the specificity of primers and probes and then further validated the specificity of primers and probes by detecting other swine respiratory pathogens using the triplex qPCR. The qPCR procedure was performed using Applied Biosystems QuantStudio 5 real-time fluorescence quantitative PCR instrument (QuantStudio^TM^ 5, Thermo Fisher Scientific). The data were processed and analyzed for specificity and the standard curves, amplification efficiency, correlation coefficient, and slope using QuantStudio Design & Analysis software (version V1.X). The date of sensitivity, repeatability, and detection of clinical samples were analyzed, and CV was calculated using Microsoft Office 2016.

## 3. Results

### 3.1. Optimization of Triplex qPCR

**Figure 2:**
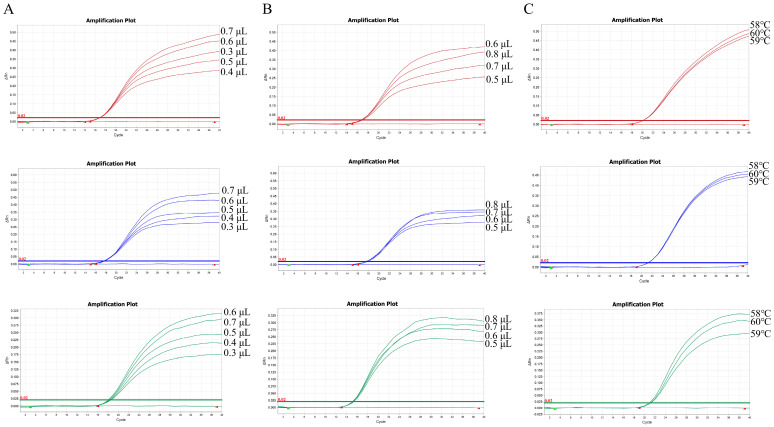
Optimization of the triplex qPCR reaction system and procedure. (**A**) Optimization of primers in the reaction system. The optimal volumes of primers were 0.3 µL for *H. parasuis* and 0.6 µL for *S. suis* serotype 2 and *P. multocida*. (**B**) Optimization of probes in the reaction system. The optimal volumes of probes were 0.6 µL for *H. parasuis* and 0.8 µL for *P. multocida* and *S. suis* serotype 2. (**C**) Optimization of annealing temperature in the reaction procedure was performed. The optimal annealing temperature was 58 °C.

Optimization of the reaction conditions of the primer, probe concentrations, and annealing temperature for triple qPCR using 1 × 10^7^ copies/µL of standard plasmid was performed as a template. The results indicated that the optimal volumes of primers for *H. parasuis*, *S. suis* serotype 2, and *P. multocida* are 0.3 µL (final concentration 150 nM), 0.6 µL (final concentration 300 nM), and 0.6 µL (final concentration 300 nM) (Figure 2A), respectively. Similarly, the optimal volumes of probes for *H. parasuis*, *S. suis* serotype 2, and *P. multocida* are 0.6 µL (final concentration 300 nM), 0.8 µL (final concentration 400 nM), and 0.8 µL (final concentration 400 nM) (Figure 2B), respectively. The confirmed reaction system is listed in Table 2. The final optimal annealing temperature was 58 °C (Figure 2C). Under these optimization conditions, triplex qPCR has the best amplification efficiency.

### 3.2. Establishment of a Standard Curve

**Figure 3:**
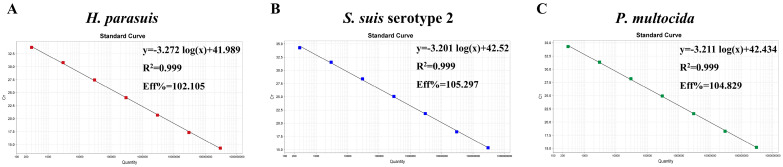
Standard curve for the triplex qPCR, the copy number of the standard plasmid ranged from 10^8^ to 10^2^ copies/µL. (**A**) The standard curve for the *H. parasuis OmpP2* gene. (**B**) The standard curve for the *S. suis* serotype 2 *gdh* gene. (**C**) The standard curve for the *P. multocida Kmt1* gene.

To establish a standard curve for the triplex qPCR, a 10-fold serial dilution of the standard plasmids was performed, utilizing concentrations ranging from 1 × 10^8^ copies/μL to 1 × 10^2^ copies/μL as templates. The respective correlation coefficients (R^2^), equation slopes, and amplification efficiencies (E%) were as follows: *H. parasuis*: 0.999, −3.272, and 102.105%; *S. suis* serotype 2: 0.999, −3.201, and 105.297%; *P. multocida*: 0.999, −3.201, and 104.829% (Figure 3). The amplification efficiencies of these three standard curves are close. Both R^2^ and E% values indicate a strong linear relationship between the initial template and Ct values, thereby validating the reliability of the qPCR standard curves.

### 3.3. Specificity Analysis

**Figure 4:**
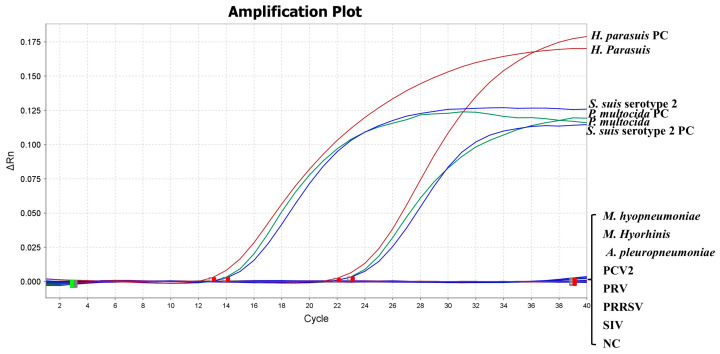
Specific analysis for the triplex qPCR assay. Ten pathogens (*H. parasuis*, *M. hyopneumoniae*, *M. hyorhinis*, *A. pleuropneumoniae*, *S. suis* serotype 2, *P. multocida*, PCV2, PRV, PRRSV, and SIV) were tested by the triplex qPCR assay. 1 × 10^7^ copies/µL standard plasmid as a positive control (PC). A nuclease-free H_2_O control was used as a negative control (NC).

Genomic DNA/cDNA of ten swine pathogens was used as templates to assess the specificity of the triplex qPCR method. The specificity analysis showed that only *H. parasuis*, *S. suis* serotype 2, and *P. multocida* have amplification curves, whereas no amplification curves were detected for other pathogens or the negative control (Figure 4). It is indicated that the triplex qPCR did not cross-react with other pathogens.

### 3.4. Sensitivity Analysis

**Figure 5:**
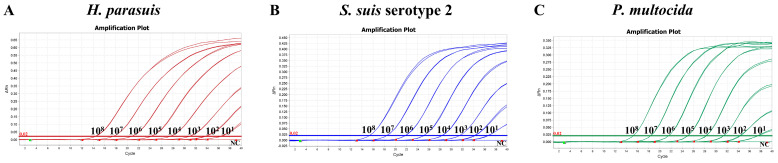
The sensitivity of the triplex qPCR assay. (**A**) Sensitivity for the *H. parasuis OmpP2* gene. (**B**) Sensitivity for the *S. suis* serotype 2 *gdh* gene. (**C**) Sensitivity for the *P. multocida Kmt1* gene.

To determine the sensitivity, 10-fold serial dilutions of standard plasmids were introduced into the amplification system. The results indicated that the LoD of the assay for *H. parasuis*, *S. suis* serotype 2, and *P. multocida* was 1 × 10^1^ copies/μL (Figure 5). Further refinement was conducted by performing a 2-fold dilution to adjust the concentration from 1 × 10^2^ copies/μL to 50 copies/μL, and plasmids at 100, 50, and 10 copies/μL were used as templates in 24 replicates. An amplification efficiency of 80% was achieved for plasmids with a Ct value of less than 40 cycles, establishing the latter as the lowest detection limit (Table 3). When the plasmid concentration was 50 copies/μL, the amplification efficiency of *H. parasuis*, *S. suis* serotype 2, and *P. multocida* could reach more than 80%, and the Ct value was approximately 32–36. Results became unreliable when the Ct value exceeded 36. Consequently, a Ct value of 36 was set as the positive threshold, indicating a positive result for Ct ≤ 36 and a negative result for Ct > 36 or no Ct.

### 3.5. Repeatability Analysis

The standard plasmids were serially diluted 10-fold, ranging from 1 × 10^6^ to 1 × 10^3^ copies/μL, and used as templates for repeatability experiments. The CVs were calculated based on the average Ct values of three replicates at each concentration. The intra-assay CVs ranged from 0.10% to 0.44%, while the inter-assay CVs ranged from 0.11% to 0.56%, both below 1% (Table 4).

### 3.6. Clinical Sample Detection

To evaluate the applicability of the method, 54 throat swab samples and 54 nasal swab samples were tested by the triplex qPCR method. The results showed that the positive rates for *H. parasuis*, *S. suis* serotype 2, and *P. multocida* in 54 throat swab samples were 96% (52/54), 100% (54/54), and 7% (4/54), respectively. For 54 nasal swab samples, the corresponding positive rates were 100% (54/54), 78% (42/54), and 4% (2/54). To verify the accuracy of the triplex qPCR method, the same clinical samples were also tested by nested PCR (GB/T34750-2017) [27] for *H. parasuis*, PCR (GB/T 19915.9-2005) [28] for *S. suis* serotype 2, and PCR (NY/T 564-2016) [29] for *P. multocida*. The positive rates for 54 throat swab samples showed that *H. parasuis*, *S. suis* serotype 2, and *P. multocida* were 93% (50/54), 28% (15/54), and 2% (1/54), respectively, while for 54 nasal swab samples, they were 98% (53/54), 2% (1/54), and 0% (0/54) (Table 5). Notably, the positive samples detected by both methods were consistent, and the sensitivity of the triplex qPCR method was observed to be higher than that of conventional PCR methods.

## 4. Discussion

In recent years, respiratory diseases have emerged as a health concern within the global swine industry, with complex microbial infections posing significant economic threats. Specifically, *H. parasuis*, *S. suis* serotype 2, and *P. multocida* are crucial pathogens for the porcine respiratory disease complex [1,31]. Individual infections often result in suppressed immunity, predisposing pigs to secondary or mixed infections, which tend to intensify inflammatory responses and even increase their mortality rate [21,31]. Furthermore, their similar clinical presentations hinder the identification of the causative pathogens through clinical observation. It is a significant threat to the development of swine herds [21]. Consequently, there is an urgent need for rapid and accurate detection methods of these pathogens.

Currently, the commonly employed molecular biology-based detection methods for *H. parasuis*, *S. suis* serotype 2, and *P. multocida* encompass traditional bacterial culturing, PCR, nested PCR, and qPCR [32,33,34]. However, the traditional bacterial culture detection process is intricate, involving multiple time-consuming steps (inoculation, separation, purification, etc.), and is prone to contamination. The PCR procedures require precise control of multiple reaction conditions; agarose gel electrophoresis is also required after the amplification procedure. The nested PCR, on the other hand, adds further complexity by incorporating additional steps, thereby heightening the risk of cross-contamination. Although loop-mediated isothermal amplification (LAMP), recombinase polymerase amplification assay (RPA), and CRISPR-Cas-based nucleic acid detection methods have emerged in recent years [19,35,36,37], LAMP and RPA suffer from limitations in quantitative analysis and susceptibility to false positives, while CRISPR-Cas detection is costly and unsuitable for high-throughput screening [38], with multiplexed detection technologies still in their infancy. Nevertheless, qPCR technology effectively mitigates these shortcomings. It offers cost-effective detection, surpasses traditional methods in terms of sensitivity and specificity, and enables multiplexed qPCR to quantitatively detect multiple target genes from different pathogens in a single-tube reaction, drastically reducing detection time, and enhancing efficiency and practicability [39]. To date, single or dual qPCR assays regarding *H. parasuis*, *S. suis* serotype 2, and *P. multocida* have been established [40,41,42,43]. When testing for the three pathogens simultaneously, using single or dual qPCR methods can result in cumbersome steps, increased time costs, and wasted sample sizes. In addition, there is a multiplex qPCR method established for 16 types of swine respiratory pathogens [44]. The 16 pathogens were divided into four units, each consisting of four pathogens, but none of the units could simultaneously detect *H. parasuis*, *S. suis* serotype 2, and *P. multocida*. If we use these methods to detect the three pathogens, it will cause problems such as increased time and reagent costs. However, there are currently no fluorescent probe assays only for simultaneous detection of all three pathogens. Consequently, we have developed a triplex qPCR assay for simultaneous detection of *H. parasuis*, *S. suis* serotype 2, and *P. multocida*. This approach not only shortens the detection time but also enhances the sensitivity and specificity of detection.

We designed three pairs of specific primers and corresponding probes targeting the conserved regions of the *OmpP2* gene of *H. parasuis*, the *gdh* gene of *S. suis* serotype 2, and the *Kmt1* gene of *P. multocida*. Each of the three probes was labeled with a distinct fluorescent reporter gene. A multi-target plasmid serving as the standard plasmid was constructed by concatenating fragments of the *OmpP2*, *gdh*, and *Kmt1* genes via restriction sites into a vector. This approach not only reduced the cost of standard plasmid construction but also minimized systematic errors compared to generating three individual plasmids. Standard curves demonstrated a strong linear correlation between Ct values and standard copy numbers. The sensitivity assays revealed detection limits of 50 copies/μL for *H. parasuis*, *S. suis* serotype 2, and *P. multocida*. This method demonstrated robust specificity, enabling the detection of *H. parasuis*, *S. suis* serotype 2, and *P. multocida* with high accuracy. Finally, 54 throat swab samples and 54 nasal swab samples were tested to verify the practicality and effectiveness of the triplex qPCR method. The results indicated that the positive samples among 54 throat swab samples for *H. parasuis*, *S. suis* serotype 2, and *P. multocida* were 52 (96%), 54 (100%), and 4 (7%). The positive samples were 54 (100%), 42 (78%), and 2 (4%) among 54 nasal swab samples. Meanwhile, the accuracy and stability of the method were validated by comparing it with nested PCR of *H. parasuis* (GB/T34750-2017) [27], PCR of *S. suis* serotype 2 (GB/T 19915.9-2005) [28], and PCR of *P. multocida* (NY/T 564-2016) [29] using the same samples. The results showed that the method established in this study was not only consistent with the standard method but also demonstrated higher sensitivity, as evidenced by the detection of a comparable or greater number of positive samples compared to the respective standard methods. Above all, it is evident that the infection rate of *H. parasuis* and *S. suis* serotype 2 both exceed 60%, whereas the infection rate of *P. multocida* is below 10%. The results indicate that the presence of *H. parasuis* and *S. suis* serotype 2 infections causes greater numbers than *P. multocida* in the Heilongjiang area. Co-infections with two or more of these pathogens were also prevalent, potentially enhancing the risk of secondary infections by other pathogens and exacerbating these diseases.

## 5. Conclusions

In conclusion, the triplex qPCR assay that was established in this study enables rapid and accurate detection of *H. parasuis*, *S. suis* serotype 2, and *P. multocida*. It can be utilized in a high-throughput mode in microbiological diagnostic laboratories, yielding results within an hour. It also provides an effective tool for microbial quality control in SPF pigs.

## Figures and Tables

**Table 1:** Primers and probes used in this study.

Name	Sequence (5′-3′)	Product Size (bp)	Reference
HPS-P2-F	GGTCTTAAATATGTCAACGCTCCA	108	This study
HPS-P2-R	CGCCAGTTCTTACGAAGTCAA		
HPS-P2-P	FAM-TGATGGTGGTCATGGTGTT-MGB		
SS-gdh-F	GAACTACGATGGCAAGGCTGAT	70	
SS-gdh-R	AGCTTGTTTGCCGTTGATCTC		
SS-gdh-P	VIC-TGCCCTTCCATGTGCGACTCAAAA-BHQ1		
Pm-Kmt1-F	GGGCRGAGTTTGGTGTGTTG	77	
Pm-Kmt1-R	GCTGAGTAATAAATAACGTCCAATCAGT		
Pm-Kmt1-P	CY5-CCAATCTGCTTCCTTGACAACGGCG-BHQ1		
HPS F1	TCGGTGATGAGGAAGGGTGA	820	Chinese national standard (GB/T34750-2017) [27]
HPS R1	TCGTCACCCTCTGTATGCAC		
HPS F2	AGGGTGGTGTTTTAATAGAGCAC	312	
HPS R2	CACATGAGCGTCAGTATTTTCC		
SS F	CCCAAGTTCAAGCCGCATTTA	495	Chinese national standard (GB/T 19915.9-2005) [28]
SS R	GAAGATTGCGAGCATTTCCTG		
Pm F	ATCCGCTATTTACCCAGTGG	460	Chinese agricultural industry standard (NY/T 564-2016) [29]
Pm R	GCTGTAAACGAACTCGCCAC		

**Table 2:** The final reaction system of triplex qPCR.

Reagent	Volume (μL)
Premix Ex Taq (Probe qPCR) (2×)	10
HPS-P2-F (10 μM)	0.3
HPS-P2-R (10 μM)	0.3
HPS-P2-P (10 μM)	0.6
SS-gdh-F (10 μM)	0.6
SS-gdh-R (10 μM)	0.6
SS-gdh-P (10 μM)	0.8
Pm-Kmt1-F (10 μM)	0.6
Pm-Kmt1-R (10 μM)	0.6
Pm-Kmt1-P (10 μM)	0.8
ROX Reference Dye	0.4
RNase-free water	1.4
Template	3
Total volume	20

**Table 3:** Positive detection rate of 100 copies/µL, 50 copies/µL and 10 copies/µL standard plasmids for 24 times.

Pathogen	Concentration (Copies/µL)	Repeat Times	Positive Number	Positive Rate	80% Positive Rate
*H. parasuis*	100	24	24	100%	>80%
	50	24	20	83%	>80%
	10	24	15	63%	<80%
*S. suis* serotype 2	100	24	24	100%	>80%
	50	24	22	92%	>80%
	10	24	19	79%	<80%
*P. multocida*	100	24	24	100%	>80%
	50	24	20	83%	>80%
	10	24	12	50%	<80%

**Table 4:** Repeatability of the triplex qPCR.

		Intra-Assay			Inter-Assay		
Pathogen	Concentration (Copies/µL)	Mean Ct Value	SD	CV (%)	Mean Ct Value	SD	CV (%)
*H. parasuis*	10^6^	21.66	0.04	0.17%	21.67	0.07	0.31%
	10^5^	25.12	0.06	0.26%	25.15	0.07	0.27%
	10^4^	28.47	0.03	0.11%	28.50	0.07	0.26%
	10^3^	31.94	0.03	0.10%	31.96	0.18	0.56%
*S. suis* serotype 2	10^6^	22.81	0.07	0.32%	22.78	0.09	0.39%
	10^5^	26.13	0.04	0.14%	26.16	0.06	0.24%
	10^4^	29.44	0.04	0.13%	29.45	0.05	0.16%
	10^3^	32.71	0.14	0.44%	32.67	0.18	0.54%
*P. multocida*	10^6^	22.48	0.07	0.32%	22.56	0.11	0.50%
	10^5^	25.92	0.03	0.12%	25.95	0.03	0.11%
	10^4^	29.15	0.03	0.11%	29.21	0.07	0.23%
	10^3^	32.39	0.10	0.32%	32.39	0.16	0.49%

**Table 5:** Detection of clinical samples.

Methods	Pathogen	Throat Swab Samples			Nasal Swab Samples		
		Number	Positive/Total Number	Positive Rate	Number	Positive/Total Number	Positive Rate
Triplex qPCR (in this study)	*H. parasuis*	54	52/54	96%	54	54/54	100%
	*S. suis* serotype 2		54/54	100%		42/54	78%
	*P. multocida*		4/54	7%		2/54	4%
nested PCR (GB/T34750-2017) [27]	*H. parasuis*		50/54	93%		53/54	98%
PCR (GB/T 19915.9-2005) [28]	*S. suis* serotype 2		15/54	28%		1/54	2%
PCR (NY/T 564-2016) [29]	*P. multocida*		1/54	2%		0/54	0%

## Data Availability

The original contributions presented in the study are included in the article, further inquiries can be directed to the corresponding authors.

## Supplementary Materials

The following supporting information can be downloaded at: https://www.mdpi.com/article/10.3390/microorganisms12102017/s1, Table S1: Specific information of the standard plasmid synthesis fragment; Table S2: Reaction mixture and protocol for nested PCR (GB/T34750-2017), PCR (GB/T 19915.9-2005) and PCR (NY/T 564-2016).
