# Supplementary material for: Development of a Triplex qPCR Assay Based on the TaqMan Probe for the Detection of Haemophilus parasuis, Streptococcus suis Serotype 2 and Pasteurella multocida
Source: Microorganisms. 2024 Oct 5;12(10):2017. doi: 10.3390/microorganisms12102017 (PMC11509477; doi:10.3390/microorganisms12102017)
Supplement: Supplementary file 1 [file microorganisms-12-02017-s001.zip › microorganisms-3206843-supplementary.pdf]

Table S1 Specific information of the standard plasmid synthesis fragment

Name	Sequence(5'-3')	Length(bp)	5'digestive site	3'digestive site
HPS-Omp P2	TTAATAAGAAAGACAAAGATGGTGTATACTTTGGTCTTAAATATGTCAACGCTCCATTTACT<br>GTAGCTGTTGATGGTGGTCATGGTGTTGAAAAAACAGGTAATGTTAAAGAGAAAATTGACT<br>TCGTAAGAACTGGCGCAAGATTTGATGTTACTCCAAAATCTGGCGTGTATGGAAACTACTCT<br>TATGGTACTTACAAAGATAAAGCTTACAAAGCAACAGCTCATCAATTCATGTTAGGTGCAG<br>ACTATAAATTACATAAACAAGTTGTTACCTTTGTTGAAGGTCGTTTAATCAAGAACAAAGAC<br>AGTAAT	314	XhoI	BamHI
SS2-gdh	CAGCTGTAGGTCCTTATAAAGGCGGTCTTCGCTTCCACCCAACTGTAAACCAATCCATCTTG<br>AAGTTCCTCGGTTTTGAGCAAATCTTCAAAAACGTCTTGACTGGTCTTCCAATCGGCGGTGG<br>TAAAGGTGGTTCAGACTTTGATCCTAAAGGAAAAACTGATGCTGAAATCATGCGCTTCTGC<br>CAAAGCTTCATGACTGAATTGCAAAAACACATCGGACCTTCACTTGACGTCCCTGCTGGTG<br>ACATCGGTGTCGGTGGTCGTGAGATCGGTTACATGTACGGTCAATACAAACGCCTCCGCCA<br>GTTTGATGCAGGTGTCTTGACTGGTAAACCTCTTGGCTTCGGTGGTTCATTGATCCGCCCAG<br>AAGCAACTGGTTGTTTGACAGAATACGCTGCAGAAAAATCAACTGCTAAGTACTTCAAAGG<br>TTCTGTATGGAACTACGATGGCAAGGCTGATATTGCCCTTCCATGTGCGACTCAAAATGAGA<br>TCAACGGCAAACAAGCTGCTGCCCTTGTAAAAAATGGCGTGTACTGTGTGGCTGAAGGTGC<br>CAACATGCCATCTGACCTTGATGCCATCAAAGTCTACAAGGAAAATGGCGTTCTCTACGGA<br>CTCGCAAAAGCTGCCAACGCTGGTGGTGTAGCTGTATCTGCCCTTGAAATGAGTCAAAACA<br>GCCTTCGCTTGTCATGGACTCGTGAAGAAGTAGACGGCCGTCTTAAAGACATCATGGCCAA<br>CATCTTCAACACAGCCAAAGAAACTGCTGAAAAATACGACCTTGGTACAGACTACCTTGCA<br>GGTGCTAACATCGCAGCCTTTGAACAAATTGCGGATAGCATGATTGCCCAAGGTTTGGTAT<br>AA	860	SacI	SalI
Pm-Kmt1	ATCCGCTATTTACCCAGTGGGGCGGTGCGAATGAACCGATTGCCGCGAAATTGAGTTTTATG<br>CCACTTGAAATGGGAAATGGCATTATTTTATGGCTCGTTGTGAGTGGGCTTGTCGGTAGTCT<br>TTTATTTGGCTTGTGGCAAAGAAAAGCACAGTTTTGTTGGGCRGAGTTTGGTGTGTTGAGCC<br>AATCTGCTTCCTTGACAACGGCGCAACTGATTGGACGTTATTTATTACTCAGCTTATTGTTAT<br>TTGCCGGTTTATATTTCCTTGTCAGTCTGATTTATCAATATTTCCATGTTGAGTTACGTTTCTT<br>ATGGCCATTATTGAAGCCATTAACGGCAGAGCGGTTTAATTTATTTATCGTGTATTGGTTAC<br>CTATTTTAGTCTTTTTCTTCGTGTTCAACGGTTTGATCGTGTCAGTCCAAATGAAACAAAAA<br>GTGGCGAGTTCGTTTACAGC	457	KpnI	NotI

Table S2 Reaction mixture and protocol for nPCR (GB/T34750-2017), PCR (GB/T 19915.9-2005) and PCR (NY/T 564-2016)

Standard	Pathogen	Method	Reagent	Volume (μL)	Protocol
The nested PCR (GB/T34750-2017) reaction mixture and protocol	*H. parasuis*	Outer PCR	2xTaq Master Mix	12.5 μl	pre-denaturation at 94°C for 2 min<br>followed by 30 cycles contain:<br>denaturation at 94°C for 30 s<br>annealing at 58°C for 30 s<br>extension at 72°C for 55 s<br>final extension at 72°C for 10 min.
			HPS F1(10 μM)	1 μl	
			HPS R1(10 μM)	1 μl	
			nuclease-free H2O	8.5 μl	
			Template	2 μl	
			Total volume	25 μl	
		Nested PCR	2xTaq Master Mix	12.5 μl	pre-denaturation at 94°C for 2 min<br>followed by 30 cycles contain:<br>denaturation at 94°C for 30 s<br>annealing at 58°C for 30 s<br>extension at 72°C for 30 s<br>final extension at 72°C for 10 min.
			HPS F2 (10 μM)	1 μl	
			HPS F2 (10 μM)	1 μl	
			nuclease-free H2O	8.5 μl	
			Outer PCR product	2 μl	
			Total volume	25 μl	
The PCR (GB/T 19915.9-2005) reaction mixture and protocol	*S. suis* serotype 2		2xTaq Master Mix	10 μl	pre-denaturation at 94°C for 2 min<br>followed by 30 cycles contain:<br>denaturation at 94°C for 30 s<br>annealing at 56°C for 30 s<br>extension at 72°C for 40 s<br>final extension at 72°C for 10 min.
			SS F (10 μM)	1 μl	
			SS R (10 μM)	1 μl	
			nuclease-free H2O	6 μl	
			Template	2 μl	
			Total volume	20 μl	
The PCR (NY/T 564-2016) reaction mixture and protocol	*P. multocida*		2xTaq Master Mix	10 μl	pre-denaturation at 94°C for 2 min<br>followed by 30 cycles contain:<br>denaturation at 94°C for 30 s<br>annealing at 55°C for 30 s<br>extension at 72°C for 40 s<br>final extension at 72°C for 10 min.
			Pm F (10 μM)	1 μl	
			Pm R (10 μM)	1 μl	
			nuclease-free H2O	6 μl	
			Template	2 μl	
			Total volume	20 μl	
